# Cardinality as a highly descriptive feature in myoelectric pattern recognition for decoding motor volition

**DOI:** 10.3389/fnins.2015.00416

**Published:** 2015-10-29

**Authors:** Max Ortiz-Catalan

**Affiliations:** ^1^Department of Signals and Systems, Chalmers University of TechnologyGothenburg, Sweden; ^2^Centre for Advanced Reconstruction of Extremities, Sahlgrenska University HospitalGothenburg, Sweden; ^3^Integrum ABGothenburg, Sweden

**Keywords:** bioelectric signal processing, cardinality, electromyography, EMG, myoelectric pattern recognition, prosthetic control

## Abstract

Accurate descriptors of muscular activity play an important role in clinical practice and rehabilitation research. Such descriptors are features of myoelectric signals extracted from sliding time windows. A wide variety of myoelectric features have been used as inputs to pattern recognition algorithms that aim to decode motor volition. The output of these algorithms can then be used to control limb prostheses, exoskeletons, and rehabilitation therapies. In the present study, cardinality is introduced and compared with traditional time-domain (Hudgins' set) and other recently proposed myoelectric features (for example, rough entropy). Cardinality was found to consistently outperform other features, including those that are more sophisticated and computationally expensive, despite variations in sampling frequency, time window length, contraction dynamics, type, and number of movements (single or simultaneous), and classification algorithms. Provided that the signal resolution is kept between 12 and 14 bits, cardinality improves myoelectric pattern recognition for the prediction of motion volition. This technology is instrumental for the rehabilitation of amputees and patients with motor impairments where myoelectric signals are viable. All code and data used in this work is available online within BioPatRec.

## Introduction

Owing to the stochastic, non-stationary, and bipolar nature of myoelectric signals, statistical or amplitude-based features are commonly used to facilitate the electromyography (EMG) relationship to motor output, such as movement or force. Researchers and clinicians rely on profiles created by EMG features to investigate and diagnose neuromuscular conditions. Therefore, accurate descriptors of muscular activity extracted from EMG are of great importance for clinical practice and research.

The prediction of movement using EMG is of particular interest for the control of prosthetic limbs and exoskeletons. Pattern recognition algorithms fed by EMG features can be used to intuitively control several robotic or virtual joints by patients who have suffered from amputation (Farina et al., 2014), stroke (Lee et al., 2011), or spinal cord injuries (Liu and Zhou, 2013). EMG features that can better characterize patterns of muscular activity play a key role in this task.

In the present study, cardinality is proposed as a superior feature for myoelectric pattern recognition (MPR) of motion volition. Compared with features that are commonly found in the literature, cardinality resulted in consistently higher MPR accuracy despite variations in sampling frequency, time window length, contraction dynamics, number and type of movements (individual or simultaneous), and different pattern recognition algorithms.

The cardinality of set A—*card(A)* or #A—is simply defined as the number of unique values within set A. For example, *A* = {1, 2, 3} and *B* = {1, 1, 2, 3, 3} have both 3 as cardinality. Unlike other amplitude depending features, such as mean absolute value (*mabs*), zero crossings (*zc*), and root mean square (*rms*), cardinality is not affected by DC offsets commonly caused by the mismatch of electrode impedance. This characteristic is shared with features such as wave length (*wl*) and number of slope changes (*slpch*). On the other hand, cardinality is dependent on the precision of the units utilized (byte, word, double, etc.), and is therefore dependent on the resolution of the analog to digital conversion (ADC), which was a factor addressed in this study.

Using information theory, Farfán et al. found that the *rms* provided a higher amount of EMG information (bits) over *mabs* and difference absolute mean value (*dam*). Conversely, the variance (*var*) provided the least EMG information (Farfán et al., 2010). Relatively complex features such as fractal dimension (*fd*) and maximum fractal length (*mfl*) have been found to improve MPR accuracy over conventional *rms, mabs, wl*, and *var* (Arjunan and Kumar, 2010). Although the decoding task in the latter study was limited to four classes using four electrodes, *mfl* continue to show higher accuracy with only a single electrode. Similarly, sample entropy was found to outperform 49 other time and frequency domain features in MPR for a single subject (Phinyomark et al., 2013). An untested feature in the previous study was rough entropy (*ren*), which Zhong et al. found produced higher accuracy than sample, wavelet, and approximate entropy (Zhong et al., 2011). As far as can be ascertained, cardinality has not been previously used as an EMG feature for MPR, and here it was found to outperform the aforementioned features.

In 1991, Hudgins et al. introduced a set of features formed by *mabs, wl, slpch, zc*, and *dam* (Hudgins et al., 1991). Two years later, they replaced *dam* by the *mean absolute value slope* (Hudgins et al., 1993). These two features were ultimately dropped to form what is currently known as the Hudgins set (*mabs, wl, slpch*, and *zc*); this is also known as the time domain (TD) set because all features are extracted from the myoelectric signal over time, as opposed to frequency. Although, no formal demonstration has been given for these features to form an optimal set, and other sets have been shown to outperform it (Oskoei and Hu, 2008; Ortiz-Catalan et al., 2012; Scheme and Englehart, 2014), the Hudgins set has been used in a considerable number of MPR studies, which makes it the most valuable set for benchmarking (review in ref., Ortiz-Catalan et al., 2013). In the present study, cardinality was found to outperform each feature within the Hudgins set, as well as to improve the accuracy when individually replaced for each feature in the set.

## Methods

### Data sets

Two data sets recorded from healthy subjects were used in this study. The first was individual movements (*IM* data): 20 subjects, 4 EMG channels, 14 bits ADC, 11 classes (hand open/close, wrist flexion/extension, pro/supination, side grip, fine grip, agree, or thumb up, pointer or index extension, and rest) (Ortiz-Catalan et al., 2013). The second was simultaneous movements (*SM* data): 17 subjects, 8 EMG channels, 16 bits ADC, 27 classes (hand open/close, wrist flexion/extension, pro/supination, and all their possible combinations) (Ortiz-Catalan et al., 2014). In both data sets, the electrodes were disposable Ag/Ag (Ø = 1 cm) in bipolar configuration (2 cm inter-electrode distance). The electrode pairs were equally spaced around the most proximal third of the forearm. The first channel was placed along the extensor carpi ulnaris, and the positive terminal of the amplifier was connected to the most proximal electrode. Both data sets are available online with detail demographics and acquisition as part of BioPatRec (Ortiz-Catalan et al., 2013), and this work has been approved by the Västra Götalandsregionen ethical committee (769–12).

### Signal acquisition, processing and classifiers

The subjects were guided by BioPatRec to perform each movement 3 times during 3 s (contraction time) with 3 s of relaxation time. The contraction time started from the moment when the subject was requested to execute a movement and continued until relaxation was prompted by the computer. Since there is a delay from request to volitional movement, as well as potential anticipatory relaxation, the beginning and end of the predefined contraction time were discarded based on a contraction time percentage (cTp). Previous work with this platform found that cTp equal to 70% eliminates silent periods while keeping the dynamic part of the contraction, while 40% will only capture the static part of the contraction (Ortiz-Catalan et al., 2013).

Unless stated otherwise, the time window length was 200 ms with a time increment of 50 ms, and the sampling rate was 2000 Hz. Features were extracted from each time window and then divided in sets for training (40%), validation (20%), and testing (40%). The accuracy was computed from the classification of the testing set only. The testing set was unseen by the classifier during training and validation. The feature vectors were randomly assigned to each set before the classifier was trained, and this was repeated 10 times per subject as cross-validation. Accuracy mean values per movement were extracted from the cross-validation per subject.

Two of the most commonly used algorithms for MPR—Linear Discriminant Analysis (LDA) (Krzanowski, 1988) and Multi-Layer Perceptron (MLP) (Haykin, 1999)—were used in this study as implemented in BioPatRec (Ortiz-Catalan et al., 2013) (code available online). When utilizing single features, convergence by MLP was observed slower; therefore, the maximum number of training iterations allowed was increased to 400, as opposed to the 200 iterations used in previous studies using BioPatRec (Ortiz-Catalan et al., 2013, 2014).

The mean of the cross-validations (10 repetitions) per subject was averaged by movement and then analyzed using the Wilcoxon Signed-Rank test (Demsar, 2006), where the pairing was done per movement.

## Results

The results are presented in box plots where the central line represents the median value; the edges of the box are the 25th and 75th percentiles; the whiskers provide the data range; outliers are represented by “+” markers; solid markers represent the mean values; and statistical significance is shown by the “^*^” marker. Tables containing the mean, standard deviation, and statistical significance for all graphs are available in Table S1.

### MPR using single features

**Figure 1:**
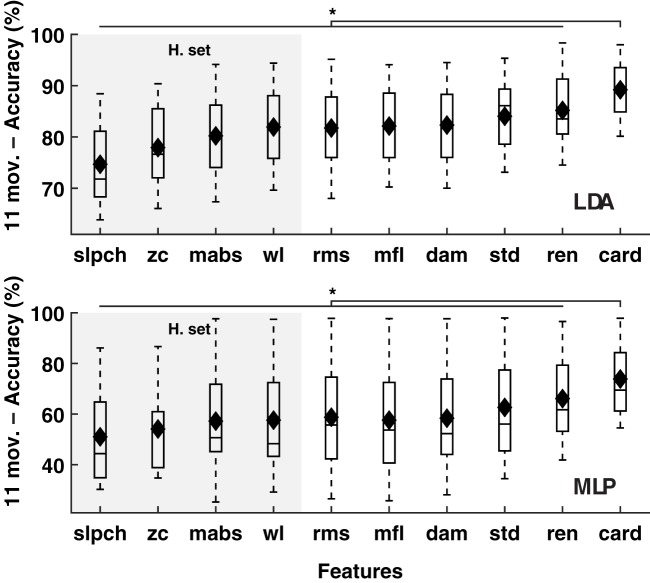
**MPR accuracy of 11 movements using single EMG features**. Accuracy of myoelectric pattern recognition (MPR) of 11 movements (20 subjects, *IM* data) using single EMG features. The LDA and MLP classifiers were employed (upper and lower insets, respectively). Cardinality outperformed previously known features, including the features from the Hudgins set (H. set). The dynamic part of the contraction was included during signal processing (cTp = 0.7). The marker “^*^” represents statistical significance at *p* < 0.01.

**Figure 2:**
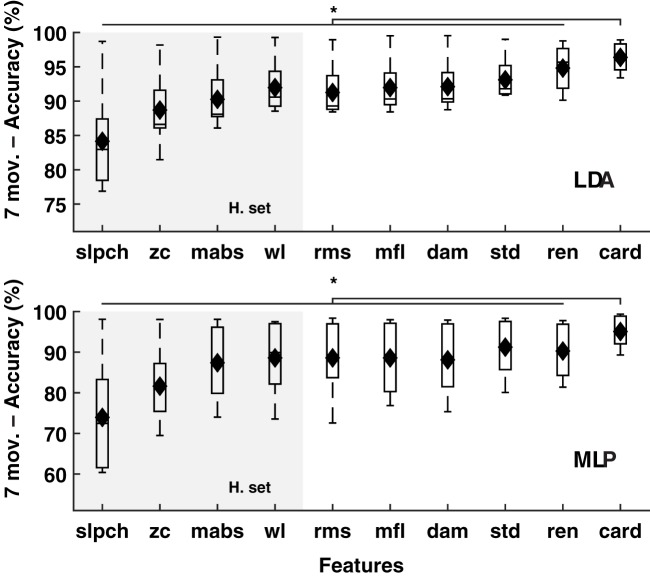
**MPR accuracy of seven movements using single EMG features**. Accuracy of myoelectric pattern recognition (MPR) of seven movements (20 subjects, *IM* data) using single EMG features. The LDA and MLP classifiers were employed (upper and lower insets, respectively). Cardinality outperformed previously known features, including the features from the Hudgins set (H. set). The dynamic part of the contraction was included during signal processing (cTp = 0.7). The marker “^*^” represents statistical significance at *p* < 0.01.

Cardinality outperformed known features in the classification of 11 hand and wrist movements (*IM* data) by LDA and MLP (*p* < 0.01); see Figure 1. Similar results were found when the number of movements was reduced to seven (hand open/close, wrist pro/supination, wrist flexion/extension, and rest), a subset for which prosthetic hardware is commercially available, and in which classification accuracy using only cardinality was over 95%; see Figure 2.

### MPR using sets of features

**Figure 3:**
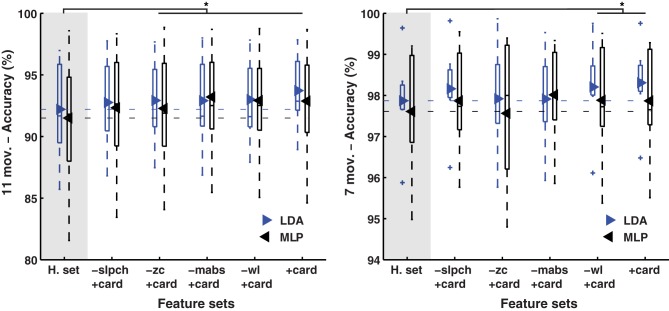
**MPR accuracy using sets of EMG features**. Accuracy of myoelectric pattern recognition (MPR) of 11 movements (left inset), and a subset of the first seven movements (right inset), using two classifiers (LDA and MLP) in 20 subjects (*IM* data). The substitution of each feature in the Hudgins set (H. set) by cardinality showed improved accuracy. The dynamic part of the contraction was included during signal processing (cTp = 0.7). The marker “^*^” represents the statistical significance at *p* < 0.05.

The classification accuracy of the Hudgins set was improved marginally (1.6%), but consistently when any of its original features were replaced by cardinality, as well as when cardinality was added to the set; see Figure 3. Improvements were found in both classifiers (LDA and MLP) when decoding 11 movements (*p* < 0.01), and to a lesser extent in the reduced subset of seven movements (*p* < 0.05). The only exception of improvement was in the latter subset using MLP and replacing *zc* (no statistically significant).

### Effect of sampling speed and time window length

**Figure 4:**
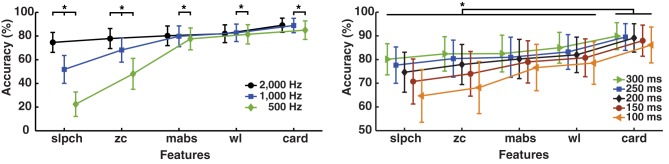
**Effect of sampling frequency and time window length**. Change in accuracy of myoelectric pattern recognition (MPR) owing to variations of sampling frequency (left inset), and the length of the time window used to extract the EMG features (right inset). Eleven movements (*IM* data) were classified using LDA and single EMG features (Hudgins' set and cardinality). The dynamic part of the contraction was included during signal processing (cTp = 0.7). The marker “^*^” represents statistical significance at *p* < 0.05.

The improvement on 11 movements discrimination by LDA using cardinality, compared with the features of the Hudgins set, was found to be consistent over variations on sampling frequency and time window length; see Figure 4. Classification accuracy using cardinality was marginally reduced by 0.2% from 2 to 1 kHz (*p* = 0.8), and by 3.9% from 2 kHz to 500 Hz (*p* < 0.01). Similar reduction was observed for *mabs* and *wl*, and more pronounced for *slpch* and *zc*. On average, accuracy reduction was found as the sampling frequency was reduced across all features.

Analogously, accuracy using cardinality was reduced by 0.4, 0.8, 1.9, and 3.5% from 300 to 250, 200, 150, and 100 ms, respectively (Figure 4, right inset). The rest of the features had similar or higher reduction, which allowed cardinality to maintain the highest accuracy in comparison (*p* < 0.01).

### Excluding the dynamic portion of muscular contraction

**Figure 5:**
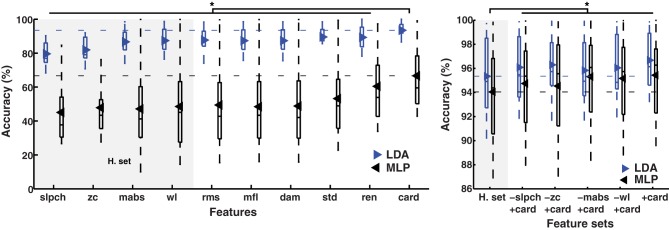
**MPR accuracy using only static contractions**. Accuracy of myoelectric pattern recognition (MPR) of 11 movements (*IM* data) using two classifiers (LDA and MLP). Cardinality outperformed previously known features, including the features from the Hudgins set (H. set), individually (left inset) and as part of the H. set (right inset). Only the static part of the contraction was included during signal processing (cTp = 0.4). The marker “^*^” represents statistical significance at *p* < 0.05.

Cardinality was also found to improve the classification accuracy of 11 movements when only considering the static portion of the contraction (cTp = 0.4); that is, completely removing the initiating isotonic portion. The improvement was consistent when using single features, as well as by modifying the Hudgins set; see Figure 5. When using single features, cardinality increased the classification accuracy by 6.1 and 18.1% for LDA and MLP (*p* < 0.01), respectively, when compared to *wl*. It is noteworthy that *wl* was found as the best performing feature in the Hudgins set, which is agreement with previous work (Scheme and Englehart, 2014).

Modifying the Hudgins set improved the classification accuracy to a modest maximum of 1.3 and 1.4% by LDA and MLP, respectively (*p* < 0.01), producing classification accuracies of 96.7% (±2.5%) by LDA, and 95.4 (±3.5%) by MLP. These represent an average classification improvement of 2.7% by removing the transient portion of the contraction; however, this is known to be detrimental for real-time performance (Hargrove et al., 2007).

### Effect of ADC resolution on MPR

**Figure 6:**
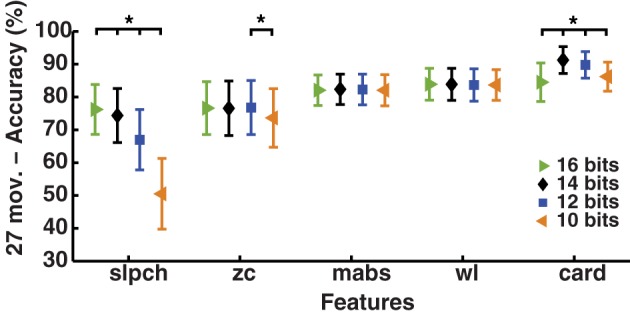
**Effect of ADC resolution on MPR accuracy**. Change in accuracy of myoelectric pattern recognition (MPR) owing to variations on the resolution of the analog-to-digital conversion (ADC). Simultaneous movements of three degrees of freedom (27 individual movements—*SM* data) were classified using LDA and single EMG features (Hudgins' set and cardinality). The dynamic part of the contraction was included during signal processing (cTp = 0.7). The marker “^*^” represents statistical significance at *p* < 0.05.

The MPR of simultaneous movements (*SM* data: 3 DoF and 17 subjects) was found to be the most accurate by LDA using cardinality; unsurprisingly, however, different ADC resolutions cause the results to vary (*p* < 0.01); see Figure 6. The highest accuracy was 91.2 ± 4.1% at an ADC resolution of 14 bits, the same resolution used to record the *IM* data. The lowest was 84.5 ± 5.9% for 16 bits, only 0.6% higher than *wl* and without statistical significance (*p* = 0.6). On the other hand, ADC resolution had practically no effect on *mabs* and *wl*, which are the Hudgins set features found with the best global performance on all previous experiments.

## Discussion

### MPR using single and sets of features

Decoding tasks can be more easily accomplished when fewer classes are to be classified, thus diminishing the effect of employing different features and classifiers. This situation was observed in this study and previous work by others (Scheme and Englehart, 2014). The difference in the classification of 11 movements between cardinality and the second best (*ren*) was 3.9 and 7.5% for LDA and MLP, respectively. This difference was reduced to 1.5 and 4.8% when the number of movements was reduced to the subset of seven (an “easier” classification task). Nevertheless, cardinality consistently produced higher classification accuracy regardless of the number of classes. In a practical sense, the studied subset of seven movements is currently the first frontier for pattern recognition systems aimed at commercially available prostheses.

Although, cardinality alone has been shown to be sufficient for producing a high classification accuracy (>95%), when added to the Hudgins set the performance was an average of 3% higher (>98%). This difference might look marginal, but one must consider that it is more difficult to increase accuracy closer to 100% than at the lower end (for example, from 70%).

It is expected that classification accuracy increases when a set of features, rather than a single one, are used to feed the classifier. This is mostly because more descriptive information is provided. Although, this work was not concerned on the search for an optimal feature set, the evaluation of the Hudgins set was performed in order to show that, in the worst case, cardinality could be substituted for any of its original features without compromising performance and, better yet, accuracy was consistently improved when cardinality was added to the set.

### Cardinality consistency as a EMG descriptor

Removing the dynamic portion of the contraction has been observed to increase the offline accuracy at the cost of real-time performance (Hargrove et al., 2007). This was done in the present study not with the intention of improving the classification accuracy, but to show that cardinality continues to outperform other features under both conditions. The same purpose was the driver for varying the length of the time window from which the features are extracted, as well as the sampling frequency at which the EMG was acquired. In both cases, cardinality showed classification improvement.

The results presented in this work corresponded to offline classification, which does not necessarily reflect real-time performance. Further work is necessary to show the prevalence of cardinality as the most descriptive feature during real-time MPR. Verification of the current results, and further real-time investigations, are facilitated by providing all the necessary code freely as done in BioPatRec (release “TRE”).

### Drawbacks of cardinality

Attention must be paid to the unit length (precision) used for signal processing prior to the computation of cardinality. Altering the dimension of the unit used for sampling (ADC resolution) to a high precision unit (for example, a double) would alienate the discrimination power of cardinality. This is because every sample value would be unique, so cardinality will always be equal to the number of samples in the time window (maximum value of cardinality).

In this application, the maximum value for cardinality can be either max(*card*) = sampling frequency (Hz) × time window (seconds), or max(*card*) = 2^(ADCbits)^. Common MPR parameters would render *card* = 2000 Hz × 0.2 ms = 400, which is considerably lower than the maximum cardinality obtained with a 10 bits resolution ADC (*card* = 1024), so the maximum value of cardinality is given by the number of samples in the time window.

A simple operation like dividing the EMG sampled values by the gain of the amplifiers, in an attempt to use the original EMG amplitude in further computations, could increase the unit precision automatically by the processing software; that is, from the original acquisition values (for example, 14 bits) to a *double*, in the case of MATLAB (Massachusetts, United States). In this study, no modifications to the originally sampled EMG values were made before extracting cardinality, as filtering was done analogously in the amplifier before the digital conversion. It is worth noting that this is normally not a problem, particularly when using microcontrollers, which are the ultimate target in this application.

The MPR accuracy at different resolutions, presented in Figure 6, suggests that cardinality would produce the highest MPR when sample values are kept within the 12–14 bits range. EMG acquisition with a higher-resolution ADC can be scaled to such a range, although this would require additional computations. The practicality of such an approach would depend on the processing hardware employed, so the use of cardinality might be restricted to particular hardware implementations.

## Conclusions

The signal features employed for myoelectric pattern recognition considerably affect the resulting accuracy. Signal processing and contraction dynamics directly affect the values of such features, and therefore the classification accuracy. Cardinality was found to consistently outperform other features despite variations in signal processing and contraction dynamics, as well as in different movement sets and classifiers. The major drawback of cardinality is its dependency on the unit precision employed, which can be overcome by scaling the signal if necessary. Overall, cardinality was found to be a highly descriptive feature of the myoelectric activity, as it yielded the highest accuracies on the decoding of motion intent.

### Conflict of interest statement

MOC was partially funded by Integrum AB during this work. All the code and data used in this work are freely available online in BioPatRec (TRE), and thus all the results presented here can be reproduced.
